# Heavy Metals in the Mainstream Water of the Yangtze River Downstream: Distribution, Sources and Health Risk Assessment

**DOI:** 10.3390/ijerph19106204

**Published:** 2022-05-19

**Authors:** Yang Jin, Quanping Zhou, Xiaolong Wang, Hong Zhang, Guoqiang Yang, Ting Lei, Shijia Mei, Hai Yang, Lin Liu, Hui Yang, Jinsong Lv, Yuehua Jiang

**Affiliations:** 1 Chinese Academy of Geological Sciences, Beijing 100037, China; jinyang@mail.cgs.gov.cn (Y.J.); liulin@mail.cgs.gov.cn (L.L.); 2 Nanjing Center, China Geological Survey, Nanjing 210016, China; zhouquanping@mail.cgs.gov.cn (Q.Z.); zhangh_nj@mail.cgs.gov.cn (H.Z.); yangguoqiang@mail.cgs.gov.cn (G.Y.); lting@mail.cgs.gov.cn (T.L.); meishijia@mail.cgs.gov.cn (S.M.); njyanghai@mail.cgs.gov.cn (H.Y.); yhui@mail.cgs.gov.cn (H.Y.); ljinsong@mail.cgs.gov.cn (J.L.); 3 Key Laboratory of Watershed Eco-Geological Processes, Ministry of Natural Resources, Nanjing 210016, China; 4 Nanjing Institute of Geography and Limnology, Chinese Academy of Sciences, Nanjing 210008, China; wangxl@njglas.ac.cn

**Keywords:** downstream, the Yangtze River, heavy metal, distribution, health risk, Monte Carlo

## Abstract

Since the mainstream of the Yangtze River lower reach is an important drinking water source for residents alongside it, it is essential to investigate the concentration, distribution characteristics and health risks of heavy metals in the water. In this study, a total of 110 water samples were collected on both the left and right banks from the upstream to the downstream. Principal component analysis (PCA) was used to determine the sources of heavy metals. Their non-carcinogenic and carcinogenic risks were studied with health risk assessment models, and uncertainties were determined through Monte Carlo simulation. Results showed that concentrations of all heavy metals were significantly lower than the relevant authoritative standards in the studied area. From the upstream to the downstream, Ni, Cu and Cr had similar concentration distribution rules and mainly originated from human industrial activities. Pb, Cd and Zn had a fluctuating but increasing trend, which was mainly due to the primary geochemistry, traffic pollution and agricultural activities. The maximum As concentration appeared in the upstream mainly because of the carbonatite weathering or mine tail water discharge. Concentrations of Zn, As, Cd and Pb on the left bank were higher than those on the right bank, while concentrations of Cu, Ni and Cr on the right bank were higher than those on the left bank. The non-carcinogenic risk index (HI) was less than 1 (except of L11), and HI on the left bank was higher than that on the right bank. The carcinogenic risk (CR) was generally larger than 1.0 × 10^−4^, CR on the right bank overall was higher than that on the left bank, and the health risk of kids was greater than that of adults. Furthermore, Monte Carlo simulation results and the actual calculated values were basically the same.

## 1. Introduction

With the continuous growth of population, the rapid urbanization and the development of modern industry and agriculture, the environmental quality of surface water has become a serious issue in many countries [1,2]. As the main environmental pollutants, heavy metals, presenting in surface water and many other media, have high toxicity, difficulty in degradation, persistence and enrichablity [3,4,5]. If these heavy metals enter the human body through the food chain and accumulate further to a certain concentration, they will be harmful to the liver, kidney, digestive system and nervous system [6,7,8], which could seriously threaten health and even cause irreversible damage [9]. Therefore, heavy metal pollution in rivers has been intensively studied recently [10,11,12,13,14,15].

The Yangtze River, as the world’s third longest and China’s largest river, is an important drinking water resource for coastal towns. It is not only the golden east–west waterway of China as the important driving force of the social and economic development but also has an important strategic position in the overall socio-economic development [16,17]. Because of the developed industry and agriculture and the dense population within the downstream of the Yangtze River, its heavy metal pollution is particularly widespread and serious, although its water quality has been significantly improved recently [18]. For example, Zhang et al. (2017) [19] found that the heavy metal concentration in the Anhui and Jiangsu sections of the Yangtze River was similar to that in Anqing and Jingjiang, which was greater than that in Nanjing and Tongling, which in turn was greater than that in Wuhu. Niu et al. (2020) [20] carried out a meta-analysis on heavy metal data in sediments from Taihu Lake during the period of 2000–2018 and pointed out that industrial pollution was the main source of heavy metals in sediments from Taihu Lake. Shan et al. (2008) [21] evaluated the characteristics and ecological risk of heavy metals in different-source sediments from the Yangtze River and found that the hazard degree of different sources showed the following order from highest to lowest: ports, industry, mines and municipal activities. Additionally, over the past few decades, the harm caused by environmental pollutants to human health has attracted more and more attention [22]. Ever since the U.S. Environmental Protection Agency for the first time had a quantitative description about the health risk assessment in 1980, the assessment model has been widely used in water and soil pollution assessment on heavy metals, etc. [23,24,25]. Yi et al., (2011) [26] analyzed heavy metals in sediment and fish from the Yangtze River and believed that the ecological risk was at an acceptable level. Gao et al. (2020) [27] studied the health risk of groundwater nitrogen in the typical karst area in East China and analyzed its uncertainty with a qualitative method. However, systematical studies on heavy metals (upstream and downstream, left and right banks) in the main waters of the Yangtze River downstream have been rarely reported.

In this study, through the comprehensive comparison between upstream and downstream, right and left banks within the studied area, (1) concentrations of Cr, As, Cd, Ni, Pb, Zn and Cu were analyzed; (2) spatial distributions of these heavy metals and their sources were discussed; and (3) their risks on human health were analyzed and explored through the health risk assessment model and the uncertainty was analyzed with the Monte Carlo simulation. These results should be an excellent reference for the water conservation and scientific management of the Yangtze River, to realize the green eco-construction of the Yangtze River.

## 2. Materials and Methods

### 2.1. Studied Area

**Figure 1:**
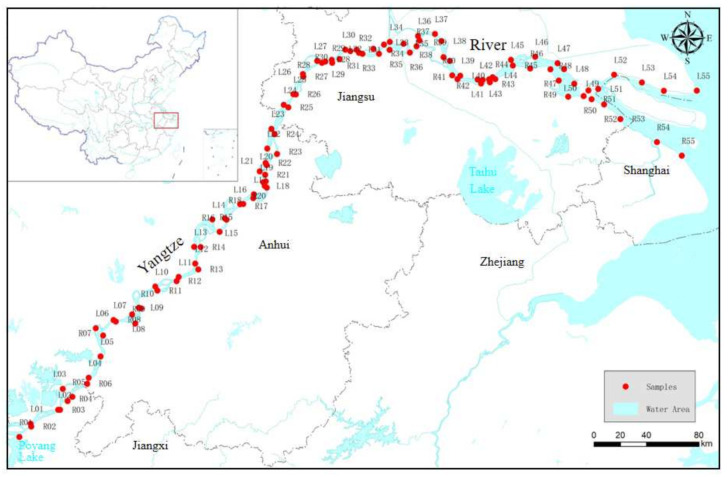
Location of studied area and distribution of sampling sites.

The Yangtze River originates from the Tibetan Plateau in the west and reaches the East Sea in the east, having a total length of about 6300 km and covering an area of about 1.8 million square kilometers. Because it runs through three steps geographically, there are significant changes in topography, geological structure and climatic background. Typically, it is divided into upper-stream, midstream and downstream with Yichang in Hubei and Hukou in Jiangxi as the boundaries, respectively. Its upper-stream has many hills, the midstream is densely covered with water networks and the downstream is dominated by plains. As shown in Figure 1, the chosen area in this study is located in the downstream that is in a subtropical monsoon climate zone with hot and humid summers, dry and cold winters, an annual average temperature of 15–20 °C and an average annual rainfall of about 1100 mm [28,29].

### 2.2. Sample Collection and Instrumental Analysis

In July of 2019, a total of 110 water samples were collected from mainstream water of upstream and downstream of the Yangtze River, including 55 samples from the left bank and 55 from the right bank (Figure 1). All samples were collected at an underwater depth of 0.5 m with previously acid-washed, 1.5 L high-density polyethylene containers, stored and transported in a low-temperature box. Each sample location was recorded with a Garmin GPS, and a description of its surrounding environment was written down. In the laboratory, the received sample was immediately filtered through a 0.45 μm Whatman filter and stored at 4 °C before analysis. An inductively coupled plasma mass spectrometer (Icap Q) was used to detect Cu, Pb, Zn, Cd and Ni in water samples. A UV–Vis spectrophotometer (TU-1950) was used to detect Cr, and an atomic fluorescence spectrometer (AFS 9600) was used to analyze As. For each sample, 3 parallel samples and 2 blank samples were analyzed. The relative error (RE) of the results was less than ±10%, and the relative standard deviation (RSD) was less than 15%, which met the U.S. EPA standard deviation (less than 30%). All data were statistically analyzed with software such as Excel 2013, SPSS 25.0 and ArcGIS 10.4.

### 2.3. Principle Component Analysis

Principal component analysis (PCA) is a mathematical tool used to reduce the complexity of the dataset and extract a small subset of latent factors called principal components (PCs) from the original set of variables [30]. Its basic principle is the process of dimension reduction. By linear transformation of numerous original variables with certain correlation, a small number of unrelated important variables are extracted to explain the main information of numerous variables. In this way, PC1 could explain more data variation than PC2, and PC2 explains more data variation than PC3, and so on. The validity of PCA is examined by the Kaiser–Meyer–Olkin (KMO) value (>0.7) and Bartlett sphericity tests (*p* < 0.001), and the correlation matrix is established based on eigenvalue decomposition [31]. PCA was widely applied to identify possible sources of heavy metal in water and sediment [32]. In this study, the professional software IBM SPSS Statistics 25.0 was used to perform factor analysis on these seven heavy metals in water, which was based on PCA.

### 2.4. Health Risk Assessment Model

Health risk assessment links environmental pollutants with human health and can quantitatively evaluate the possibility or degree of damage of physical or chemical factors to the human body under specific environmental conditions [33]. Studies have shown that heavy metals in water can cause harm to the human body mainly through three paths, including direct ingestion through hand and mouth, dermal adsorption through exposure and inhalation through breathing. With the water environment health risk assessment model recommended by the U.S. Environmental Protection Agency (U.S. EPA) [34,35], this study was mainly focused on health risks caused by carcinogenic and non-carcinogenic heavy metals that enter into adults and kids through drinking water and skin contact. Risk can be calculated with the following equations:

(1) $$Q_{\mathrm{ingestion}}=\frac{\mathrm{CW}\times \mathrm{IR}\times \mathrm{ABSGI}\times \mathrm{EF}\times \mathrm{ED}}{\mathrm{BW}\times \mathrm{AT}},$$

(2) $$Q_{\mathrm{dermal}}=\frac{\mathrm{CW}\times \mathrm{KP}\times \mathrm{SA}\times \mathrm{EF}\times \mathrm{ED}\times \mathrm{ET}}{\mathrm{BW}\times \mathrm{AT}},$$

where Q is average daily dose of heavy metals by ingestion (Q_ingestion_) and dermal absorption (Q_dermal_), unit in mg/(kg/d). C_w_ is the average concentration of heavy metal in water, in mg/L. Other parameters in the formula are shown in Table 1.

Carcinogenic risk and non-carcinogenic risk are used to determine the risk posed by the heavy metals in water. The hazard quotient (HQ, Equation (3)) reflects the potential non-carcinogenic risk, which is obtained from the comparison between the amount of ingested heavy metal through various exposure routes (direct ingestion and dermal exposure) and its response reference dose (RfD, Equation (4)). In order to evaluate the total potential non-carcinogenic risk posed by more than one pathway, the hazard index (HI, Equation (5)) was introduced, which was the sum of the hazard quotients from all the applicable pathways. If HI or HQ exceeds 1, there might be cause for concern regarding non-carcinogenic effects on human health.

(3) $$\mathrm{HQ}=\frac{Q}{\mathrm{RfD}},$$

RfD_dermal_ = RfD × ABS_GI_, (4)

(5) $$\mathrm{HI}=\sum_{i=1}^{n}\mathrm{HQ},$$

Equation (6) is applied to evaluate the carcinogenic risk of heavy metal in water. The estimated value is used to show the possible lifetime carcinogenic risk of exposure to a carcinogenic heavy metal to the human body.

CR (Cancer Risk) = Q × CSF (Cancer slope factor),(6)

According to the list of carcinogens determined by the International Agency for Research on Cancer (IARC) of the World Health Organization (WHO), among the seven heavy metals in this study, Cr, As and Cd are carcinogens with both carcinogenic and non-carcinogenic risks to human health. Cu, Zn and Ni are non-carcinogens and may have non-carcinogenic risks. Their toxicological characteristic parameters are listed in Table 2. Up to the present, the acceptable carcinogenic risk index limit, which is recommended by the International Commission on Radiation Protection (ICRP), is 5.0 × 10^−5^. The Dutch Ministry of Housing Space Planning and Environment sets 10^−4^ as the acceptable carcinogenic risk index limit. In China, the Technical Guidelines for Risk Assessment of Contaminated Sites recommends 10^−6^ as the acceptable carcinogenic risk level.

### 2.5. Monte Carlo Simulation

As one of the powerful tools in risk analysis [41,42], Monte Carlo simulation has been widely used since the 1940s as a statistical technique in uncertainty assessment because threats and opportunities are both considered simultaneously, and various standard probabilities are chosen to simulate, to build the distribution of output variables through re-sampling from many input varieties [43] and to transform the uncertainty analysis into a quantitative probability. In this study, the software Oracle Crystal Ball, combined with Microsoft Excel, was used to carry out Monte Carlo simulation on the health risk uncertainty. The entire simulation included determining the best fitting distribution, defining the assumption unit and prediction unit, determining the number of simulations and starting the simulation operation. The fitting distribution mainly included 21 commonly used mathematical distributions, such as normal distribution, triangular distribution, beta distribution, lognormal distribution, and Gumbel distribution. Fitting test methods mainly included the Anderson–Darling test, Kolmogorov–Smirnov test and chi-square test. In an excellent fitting, these calculated values were <1.5, >0.5 and <0.03, respectively.

## 3. Results and Discussion

### 3.1. Concentration of Heavy Metals in Water

As listed in Table 3, in the mainstream water of the Yangtze River downstream, the average concentrations of heavy metals were in the following order: Cd < Ni = Cr < Pb < Cu < As < Zn, and Zn had the largest concentration range of 2.40–44.60 μg/L. Their maximum concentration values were all within China’s “Standards for Drinking Water Quality” (GB 5749-2006) and “Environmental Quality Standards for Surface Water” (GB3838-2002) Class I standards, WHO standards, and U.S. EPA standards. Their coefficients of variation (CVs) were medium and followed the order from high to low as Cu, Ni, Cr, Cd, As, Zn and Pb.

**Figure 2:**
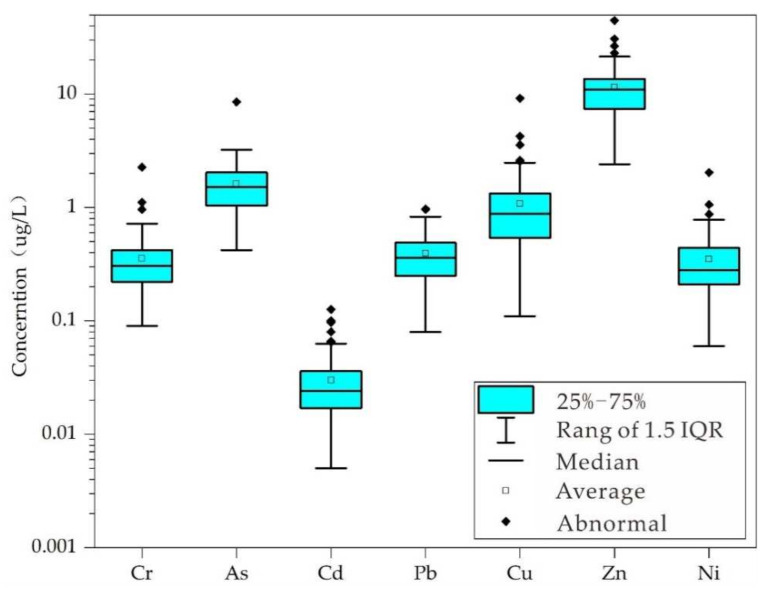
Boxplot of heavy metal concentration.

The average concentration of every studied heavy metal in these waters was higher than the median, indicating that the number of low-concentration water samples was dominant in these collected 110 samples. All abnormal values were extremely large. Cd, Cu and Zn all had four high-value points, accounting for 3.64%. Cr and Ni had three high-value points, respectively, accounting for 2.73%. As and Pb had one high-value point, respectively, accounting for 0.91% (Figure 2).

Compared with concentrations of heavy metals in other waters (Table 4), heavy metals in the studied area were generally at a low level. In China, concentrations of Cu and Cd in the studied area were comparable to that of the Pearl River, that of Zn was about four times that of the Pearl River, that of As was slightly higher than that of the Luan River, that of Cd was about 1/2 of that of the Jiulong River and concentrations of other heavy metals were significantly lower than those of other rivers in China. Therefore, heavy metal concentrations in the studied area were similar to those of the Pearl River. Additionally, except that the Pb concentration was slightly higher than that of the Tigris River in Turkey and the Zn concentration was higher than that of the Catalan River in Spain, concentrations of other heavy metals were all lower in the studied area than other foreign rivers, although they were similar to those of the Subarnarekha River in India and the Pardo River in Brazil.

### 3.2. Spatial Distribution and Source Analysis of Heavy Metals

**Figure 3:**
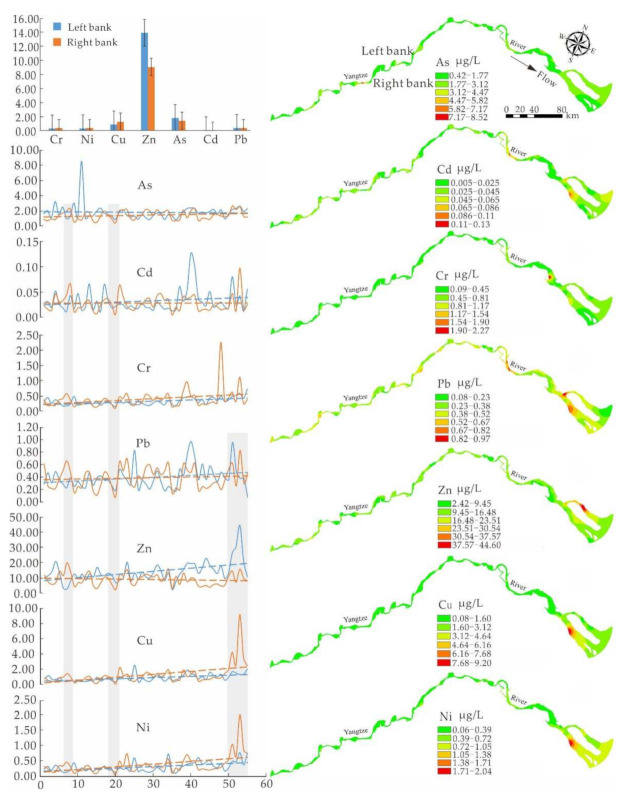
Distribution of heavy metal concentrations in water within the studied area.

According to the spatial distribution of heavy metal concentrations in the studied area (Figure 3), from upstream to downstream, concentration distributions of Ni, Cu and Cr were similar with a small upward trend. Concentration distributions of both Pb and Cd had a relatively large upward trend, and their peaks were concentrated in the midstream and downstream of the studied area. Zn had a relatively large fluctuation with an upward trend on the left bank but a downward trend on the right bank, and a peak in the downstream of the studied area. The peak of As appeared in the upstream of the studied area with a downward trend on the left bank, while there was a small upward trend on the right bank. These tendencies may be related to human activities and changes in nature responding to that. In recent decades, over 50 thousand dams were built along the Yangtze River [52]. With the construction and operation of major water conservation projects in the upstream region of the Yangtze River, equilibrium conditions between water and sand were changed greatly. Moreover, clean-water discharge exacerbated channel erosion [53], and heavy metals deposited in sediment could be transferred into water and accumulated in the downstream by the influence of water flow [54].

Results showed that the average concentrations of Zn, As, Cd and Pb were higher on the left bank than those on the right bank, while those Cu, Ni and Cr were higher on the right bank. Concentrations of heavy metals in water on the left and right banks were consistent around the 20th sampling site with a “V” trend, and the minimum concentrations of these seven heavy metals on the right bank. Concentrations of heavy metals in the water body around sampling sites 7–9 showed an irregular mirror-image relationship, that is irregular with a “V” trend on the left bank and an inverted “V” on the right bank. Trend tails of Ni, Cu, Zn and Pb on the left and right banks were consistent with “double peaks and double valleys”, which might be related to the discharge of local urban sewage.

The KMO and Bartlett test results were 0.724 (>0.7) and 0.000 (<0.001) in the study, respectively, indicating that the PCA was appropriate for analyzing the source of heavy metals [55]. In order to better understand the actual meaning of each factor, the maximum variance method was used to rotate each factor to obtain the rotated component matrix as listed in Table 5. A total of three principal components (Eigenvalues > 1), which were extracted from the seven heavy metals in the studied area, revealed 78.368% of influencing factors in the water body. Principal component 1 (PC1), principal component 2 (PC2) and principal component 3 (PC3) had total factors of 51.428%, 15.653% and 11.287%, respectively, which effectively showed most of the information from the original data.

As listed in Table 5, PC1 had a higher positive loading in the Cr, Ni, and Cu with a significantly high variation coefficient, indicating a similar origin. According to the reported results, the development of large/medium cities along the Yangtze River, and their industrial and agricultural developments, which were affected by the distribution of stepped landforms, were mainly within its midstream and downstream [56]. Within the downstream of this studied area, especially in Shanghai and the south of Jiangsu with dense industries and high population, heavy metals in PC1 might be related to human activities, and chemical fertilizers and pesticides used in agricultural activities had a smaller impact on Cr and Ni than the soil itself [57]. Therefore, it could be believed that heavy metals in PC1 mainly came from industrial activities with peak values in the downstream of the studied area, which was consistent with that from Wang et al. [58].

PC2 has relatively large positive loadings in Zn, Cd and Pb with lower variation coefficients than those of Cr, Ni, Cu in PC1, indicating that there might be various origins. Results showed that concentrations of Zn, Cd and Pb in soil were affected by both parent materials and soil-forming process, which subsequently entered all tributaries with atmospheric precipitation and surface runoff [59] and then flowed into the Yangtze River. Therefore, geochemistry is one of its sources. Because automobile tires are an important source of Zn in the environment, and automobile exhaust emission is an important source of Pb, traffic pollution is believed as one of the sources of Zn and Pb. Generally, Cd is a marker element of agricultural activities [60,61], and pesticides and fertilizers have Zn and Pb [46]. Therefore, agricultural activities should also be the sources of Zn, Cd and Pb in water bodies.

The loading of As in PC3 was the largest, and it was the only heavy metal with the peak appearing in the upstream within the studied area (Figure 3), indicating that As had an independent source. Studies have shown that the concentration of As is relatively low in basic–acidic magmatic rocks but strongly enriched in carbonatites [62,63]. The weathering decomposition and soil erosion of carbonatite in the Hukou area, and mine tailwater discharge from Zongyang and Guichi should be the main source of As in the upstream water of the studied area.

### 3.3. Health Risk Assessment on Heavy Metals

Because the drinking water of about 150 million people has been affected, surface water contamination has become a great challenge for the whole world [1]. Although the water supply in northern China is mostly groundwater, surface water is dominant in the southern part with 49 urban drinking water sources in the midstream and downstream of the Yangtze River for water supply to a population of 17.26 million annually and 16 listed in the National List of Important Drinking Water Sources, including 13 in the downstream [64]. Therefore, it is of great significance to assess the health risks of heavy metals in the mainstream water of the Yangtze River lower reach.

Based on the health risk assessment model, heavy metal concentrations and all chosen parameters, the health risks of heavy metals from drinking water and dermal exposure to adults and kids in the studied area were calculated. Results showed that health risks of heavy metals in water to kids were significantly higher than those to adults, in which the non-carcinogenic risk and carcinogenic risk of kids were 1.43 and 1.41 times those of adults, respectively (Table 6). Therefore, compared with adults, kids are more susceptible to the threat of heavy metals, which is consistent with previous reports [65,66] and suggested that the children’s water safety requirements should be paid more attention.

**Figure 4:**
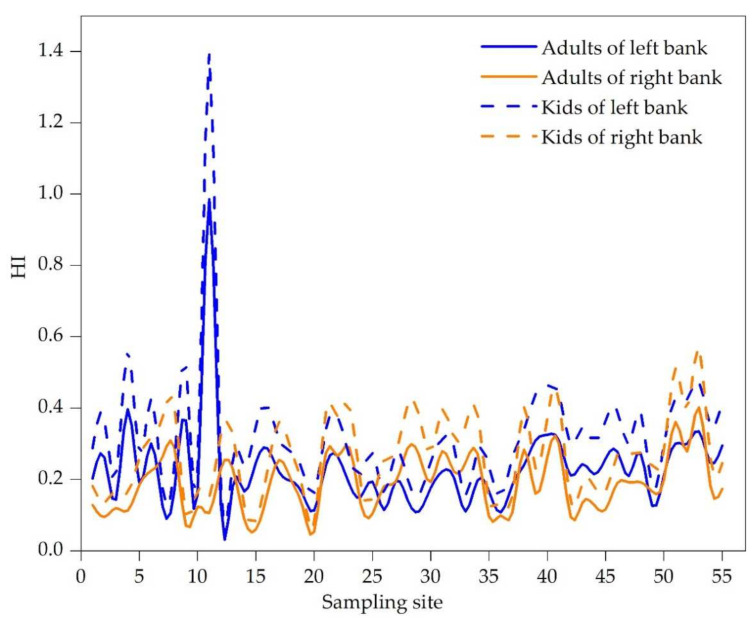
Comparison of non-carcinogenic risk index distribution between adults and kids on the left bank and the right bank.

As shown in Figure 4, from upstream to downstream, the HI values of non-carcinogenic risk in all sampling sites were less than 1.0 (except that of site L11). HI on the left bank was slightly higher than that on the right bank (HI_left_ > HI_right_). However, the non-carcinogenic risks (HQs) of Cr, Ni and Cu on the right bank were higher than those on the left bank (Table 4), indicating that these four heavy metals (Cd, Pb, Zn and As) contributed relatively large to the non-carcinogenic risk on the left bank. Especially, the HQ value of As at site 11 in the upstream was 1.37, which was higher than the acceptable limit of non-carcinogenic risk of 1.0 [38] and might have adverse effects on human health, leading to liver cancer, lung cancer, bladder cancer, kidney cancer and skin cancer [67]. These results were similar to those reported previously, indicating that As was one of the priority pollution elements in the midstream and downstream of the Yangtze River [26,68].

**Figure 5:**
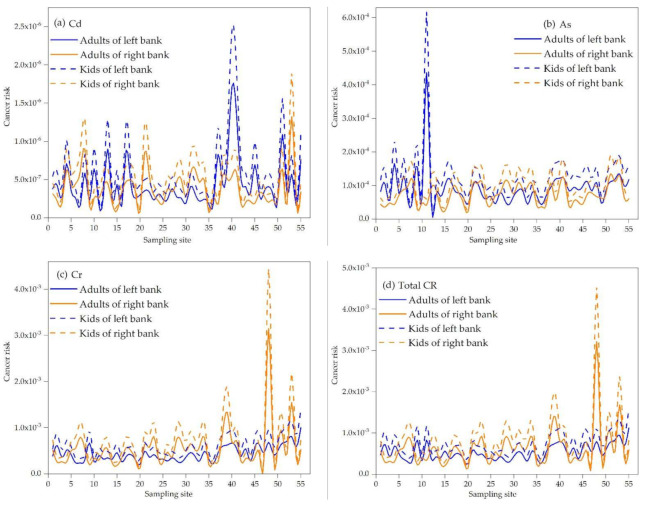
Comparison of carcinogenic risk distributions (**a**) CR value of Cd comparison between adults and kids on the left bank and the right bank; (**b**) CR value of As comparison between adults and kids on the left bank and the right bank; (**c**) CR value of Cr comparison between adults and kids on the left bank and the right bank; (**d**) total CR value comparison between adults and kids on the left bank and the right bank.

In terms of carcinogenic risk, Cd was higher on the left bank than on the right bank with a “W”-shaped fluctuation rising trend from the upstream to downstream within an order of magnitude of 10^−7^–10^−6^ (Figure 5a), which was far lower than that recommended by the International Commission on Radiation Protection and the U.S. Environmental Protection Agency (5.0 × 10^−5^ and 1.0 × 10^−4^), while it was higher in some areas than the maximum acceptable risk level recommended by the Dutch Ministry of Construction and Environmental Protection, the Royal Society of the United Kingdom and the Swedish Environmental Protection Agency (1.0 × 10^−6^). Carcinogenic risk of As was also higher on the left bank, while a first increase was followed by a decrease from the upstream to downstream, and then it fluctuated around the average value with an order of magnitude of 10^−5^–10^−4^ (Figure 5b). As shown in Figure 5c, the carcinogenic risk of Cr was different from those of Cd and As, wherein, on the right bank, it was higher with an increasing trend from the upstream to downstream and an order of magnitude of 10^−4^–10^−3^. The overall carcinogenic risks of heavy metals from the upstream to downstream in the studied area showed an increasing trend. As shown in Figure 5d, the carcinogenic risk on the right bank was generally higher, which was mainly affected by heavy metal Cr, with an order of magnitude of 10^−4^–10^−3^.

Although results in this study showed that the concentrations of heavy metals in the mainstream of the Yangtze River lower reach were relatively low and did not exceed the limit specified by the relevant water quality standards, there were non-carcinogenic risks (especially that of As) larger than 1 and carcinogenic risks (especially that of Cr) exceeding the maximum acceptable level of 1.0 × 10^−4^, which might be related to the toxicological properties of different heavy metals [39]. Therefore, health risks still require continuous attention, and it is necessary to introduce health risk assessment into water quality monitoring and water resources management, especially to strengthen health risk management in drinking water sources.

### 3.4. Analysis on the Uncertainty of Health Risk Assessments

**Figure 6:**
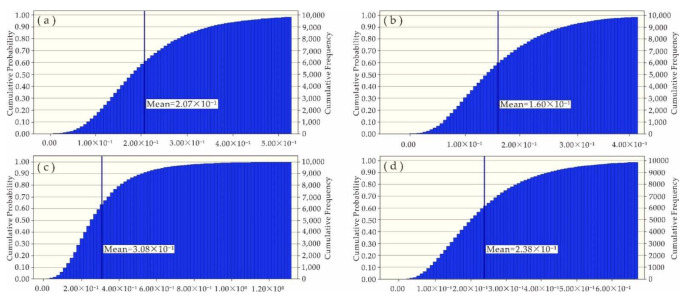
Simulated HQ (As) values of (**a**) adults on the left bank; (**b**) adults on the right bank; (**c**) kids on the left bank; and (**d**) kids on the right bank.

**Figure 7:**
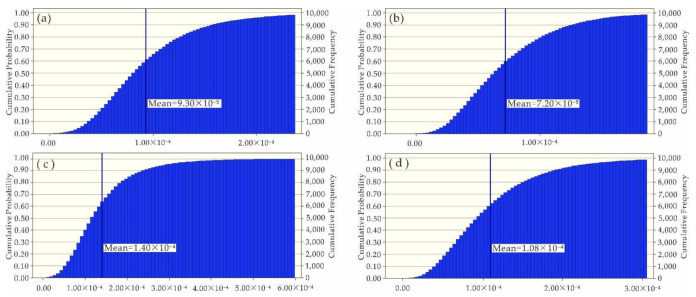
Simulated CR (As) values of (**a**) adults on the left bank; (**b**) adults on the right bank; (**c**) kids on the left bank; and (**d**) kids on the right bank.

**Figure 8:**
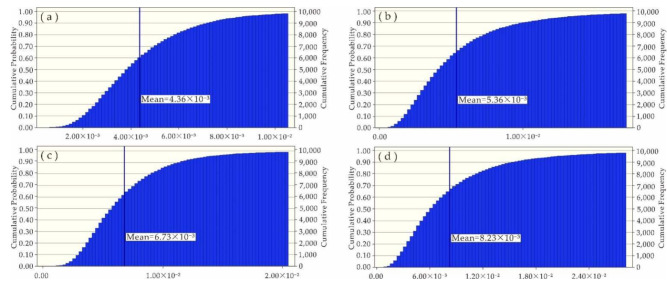
Simulated HQ (Cr) values of (**a**) adults on the left bank; (**b**) adults on the right bank; (**c**) kids on the left bank; and (**d**) kids on the right bank.

**Figure 9:**
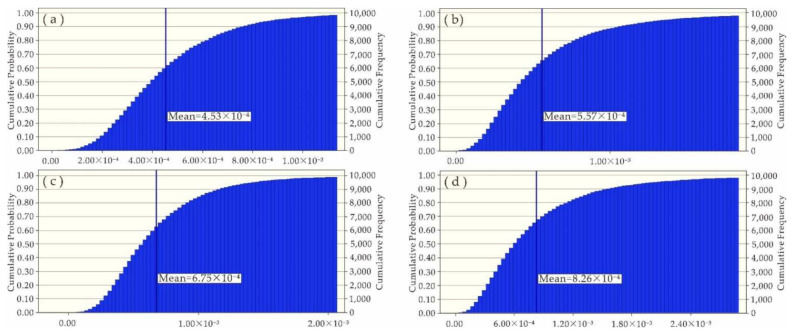
Simulated CR (Cr) values of (**a**) adults on the left bank; (**b**) adults on the right bank; (**c**) kids on the left bank; and (**d**) kids on the right bank.

Since the impacts of Ni, Zn, Cu, Pb and Cd on human health risks were at an acceptable level, this study was focused on the uncertainty analysis of health risks caused by As and Cr. With the software Crystal Ball and Microsoft Excel, Monte Carlo simulation was carried out, in which the measured concentrations of heavy metals in water samples, average daily water intake and body weight were defined as hypothetical units, but the non-carcinogenic risk (HQ) and carcinogenic risk (CR) were defined as the prediction units. After 10,000 simulation samples were performed (Figure 6, Figure 7, Figure 8 and Figure 9), the simulation results were basically consistent with the calculated ones (Table 7).

It was well known that uncertainty in health risk assessment could be caused by various stages with subjective and objective uncertainties from field sample collection, indoor test and analysis, model parameter acquisition, model applicability and assumption, exposure parameters and toxicology data. Therefore, in this study, general parameters were chosen in the calculation although it might be different from the actual situation in the studied area due to the large area with the over-complicated antagonism and synergy between various heavy metals. The exposure to heavy metals in water only included drinking ingestion and dermal exposure without the consideration of other routes such as food intake, air inhalation and other factors closely related to people’s occupational types, consumption habits, etc. Additionally, in this study, water samples were collected from the mainstream of the Yangtze River lower reach, while the actual drinking water of residents was treated water from water plants, in which concentrations of heavy metals might be lower than the values in these samples. There was a possibility of overestimating the risk of heavy metal exposure, which should be further studied in the future.

## 4. Conclusions

Quantitative assessment and overall identification of the health risk caused by heavy metals in the mainstream water of the Yangtze River downstream is vitally important for nearby inhabitants to keep healthy and for the Yangtze River to develop sustainably. Concentrations of heavy metals in the water within the studied area were far below the limit of relevant authoritative standards and at a lower level compared with those in other rivers worldwide. Average concentrations of Zn, As, Cd and Pb on the left bank were higher than those on the right bank, while those of Cu, Ni and Cr on the left bank were lower. From the upstream to downstream, concentration distributions of Ni, Cu and Cr all showed small fluctuations and upward trends, which were closely related to the intensity of human industrial activities. Pb and Cd had relatively large fluctuations and upward trends with peaks concentrated in the midstream and downstream of the studied area. Zn showed a relatively large fluctuation with a significant upward trend on the left bank, a downward trend on the right bank and the peak in the downstream, which were related to the original geochemical background, traffic pollution and agricultural activities. The peak of As appeared in the upstream with a downward trend on the left bank and a small fluctuation and upward trend, which were related to the weathering and decomposition of carbonatite and the discharge of mine tail water. Except of the sample site of L11, the non-carcinogenic risks of heavy metals in the studied area were at an acceptable level, and the risk was higher on the left bank compared to that on the right bank. However, carcinogenic risks, which were mainly affected by Cr and As, were at an unacceptable level, and the risk on the right bank was overall higher than that on left bank. Additionally, the impacts of heavy metals on the health risks on children were significantly larger than those on adults. Although the Monte Carlo simulation results were basically consistent with the actual calculated values, the uncertainty analysis considering more factors should be further studied and improved.

## Figures and Tables

**Table 1:** Heavy metal exposure parameters [9,36,37,38].

Parameter	Unit	Adult	Kid
Average body weight (BW)	kg	62.4	20.08
Average exposure time (AT)	d	10,950	4380
Exposure frequency (EF)	d/a	350	350
Average ingestion rate (IR)	L/d	2.2	1.0
Absorption factor of gastrointestinal (ABS_GI_)	dimensionless	1	1
Exposure duration (ED)	a	30	12
Dermal adsorption parameters (K_P_)	cm/h	0.001	0.001
Exposure time (ET)	h/d	0.5	0.5
Body surface area (SA)	cm^2^	16,600	9500

**Table 2:** Toxicological characteristic parameters of heavy metals [38,39,40].

Heavy Metal	Non-Carcinogenic Reference Dose (RfD)/mg·kg^−1^·d^−1^		Carcinogenic Slope Factor (CSF)/mg^−1^·kg·d	
	Drinking	Dermal	Drinking	Dermal
As	0.0003	0.000123	1.5	3.66
Cd	0.0005	0.000005	0.38	6.1
Cr	0.003	0.00006	41	41
Ni	0.02	0.02	/	/
Pb	0.0014	0.0014	/	/
Cu	0.005	0.005	/	/
Zn	0.3	0.06	/	/

**Table 3:** Statistics of heavy metal concentrations (*n* = 110).

HM	Range	Mean ± SD	China ^1^	Class I ^2^	WHO [44]	U.S. EPA (MCL) [45]	CVs
Cr	0.09–2.26	0.35 ± 0.24	50	10	50	100	69.00%
As	0.42–8.52	1.61 ± 0.89	10	50	10	10	54.85%
Cd	0.01–0.13	0.03 ± 0.02	5	1	3	5	66.36%
Pb	0.08–0.97	0.39 ± 0.18	10	10	10	15	45.80%
Cu	0.11–9.17	1.08 ± 1.02	1000	10	2000	1300	94.80%
Zn	2.40–44.6	11.49 ± 6.10	1000	50	/ ^3^	/	53.06%
Ni	0.06–2.03	0.35 ± 0.25	20	/	70	/	70.21%

^1^ Ministry of Health, P.R. China, 2007. Standards for drinking water quality (GB5749-2006). ^2^ Ministry of Ecology and Environment, P.R. China, 2002. Environmental Quality Standards for Surface Water (GB3838-2002). ^3^ “/” indicates there are no data available yet.

**Table 4:** Heavy metal concentrations in different waters in China and other countries.

River	Cr	As	Cd	Pb	Cu	Zn	Ni
Downstream of Yangtze River	0.35	1.61	0.03	0.39	1.08	11.49	0.35
Xiangjiang [12]	6.61	12.24	1.34	2.29	20.33	84.57	/
Zhujiang [13]	1.695	/	0.042	0.077	1.092	3.611	1.892
Yellow River [14]	23.19	7.3	23.19	19.51	36.27	52.46	25.11
Luan River [15]	/	1.5	0.57	4.7	1.43	/	/
Jiulong River [46]	5.411	12.393	0.077	4.467	17.853	154.893	3.989
Catalan River (Spain) [47]	2.4	2.9	1.2	2.2	1.3	1.9	2.7
Tigris River (Turkey) [48]	<5	2.35	1.37	0.34	165	37	72
Bilina River (Czech Republic) [49]	/	14.13	0.21	7.92	/	35.26	/
Subarnarekha River (India) [50]	0.47	2.13	/	/	3.35	/	2.39
Pardo River (Brazil) [51]	0.5	1.93	0.06	4.1	2.88	13.14	6.33

**Table 5:** Composition matrix after the rotation.

Heavy Metal	Principal Components		
	PC1	PC2	PC3
Cr	0.737	0.018	0.261
Ni	0.860	0.349	0.115
Cu	0.860	0.365	−0.026
Zn	0.059	0.605	0.562
As	0.182	0.135	0.898
Cd	0.230	0.845	0.079
Pb	0.317	0.821	0.190
Eigenvalues	3.600	1.096	1.007
Variance contribution rate (%)	51.428	15.653	11.287
Accumulated contribution rate (%)	51.428	67.081	78.368

**Table 6:** Health risk assessment index of heavy metals.

Property		Adult				Kid			
		Range on the Left Bank	Average on the Left Bank	Range on the Right Bank	Average on the Right Bank	Range on the Left Bank	Average on the Left Bank	Range on the Right Bank	Average on the Right Bank
Non-carcinogenic risk (HQ)	Ni × 10^−4^	1.87~13.23	5.33	1.02~34.44	6.54	2.64~18.71	7.54	1.44~48.70	9.25
	Zn × 10^−4^	2.76~51.21	16.01	2.87~22.96	10.38	3.91~72.68	22.72	4.07~32.59	14.73
	Cu × 10^−3^	2.24~17.24	5.99	0.75~62.24	8.61	3.17~24.37	8.47	1.06~88.00	12.17
	Pb × 10^−3^	1.94~23.51	9.53	2.18~20.12	9.47	2.74~33.24	13.48	3.08~28.45	13.38
	Cd × 10^−3^	1.02~11.73	2.98	0.47~9.03	2.61	1.55~17.75	4.50	0.70~13.66	3.95
	As × 10^−2^	9.55~96.90	20.50	4.78~30.02	16.22	13.53~137.19	29.03	6.76~42.51	22.97
	Cr × 10^−3^	2.01~9.64	4.23	1.21~30.27	5.24	2.95~14.18	6.21	1.77~44.52	7.71
	HI	0.11~0.98	0.23	0.05~0.40	0.19	0.15~1.39	0.33	0.07~0.57	0.27
Carcinogenic risk (CR)	Cd × 10^−7^	1.50~17.17	4.36	0.68~13.22	3.82	2.15~24.61	6.24	0.98~18.94	5.48
	As × 10^−5^	4.30~43.60	9.23	2.15~13.51	7.30	6.09~61.74	13.06	3.04~19.13	10.34
	Cr × 10^−4^	2.09~10.02	4.39	1.25~31.44	5.45	2.95~14.16	6.21	1.77~44.46	7.70
	CR × 10^−4^	2.52~14.4	5.32	1.47~32.8	6.18	3.56~20.4	7.52	2.08~46.4	8.74

**Table 7:** Simulated values versus calculated values.

Property		Non-Carcinogenic Risk (HQ)		Carcinogenic Risk (CR)	
		As	Cr	As	Cr
Adults on the left bank	Simulated value	2.07 × 10^−1^	4.36 × 10^−3^	9.30 × 10^−5^	4.53 × 10^−4^
	Calculated value	2.05 × 10^−1^	4.23 × 10^−3^	9.32 × 10^−5^	4.39 × 10^−4^
Adults on the right bank	Simulated value	1.60 × 10^−1^	5.36 × 10^−3^	7.20 × 10^−5^	5.57 × 10^−4^
	Calculated value	1.62 × 10^−1^	5.24 × 10^−3^	7.30 × 10^−5^	5.45 × 10^−4^
Kids on the left bank	Simulated value	3.08 × 10^−1^	6.73 × 10^−3^	1.40 × 10^−4^	6.75 × 10^−4^
	Calculated value	2.90 × 10^−1^	6.21 × 10^−3^	1.31 × 10^−4^	6.21 × 10^−4^
Kids on the right bank	Simulated value	2.38 × 10^−1^	8.23 × 10^−3^	1.08 × 10^−4^	8.26 × 10^−4^
	Calculated value	2.30 × 10^−1^	7.71 × 10^−3^	1.03 × 10^−4^	7.70 × 10^−4^

## Data Availability

The data that support the findings of this study are available from the corresponding author, Y.-H. Jiang (jyuehua@mail.cgs.gov.cn), upon reasonable request.
